# MicroRNA 617 Targeting SERPINE1 Inhibited the Progression of Oral Squamous Cell Carcinoma

**DOI:** 10.1128/MCB.00565-20

**Published:** 2021-05-21

**Authors:** Chunguang Zhao, Zhiyun Liu

**Affiliations:** a Department of Stomatology, The Sixth Hospital of Wuhan, Affiliated Hospital of Jianghan University, Wuhan, Hubei, China

**Keywords:** oral squamous cell carcinoma, SERPINE1, miR-617, proliferation, apoptosis, cancer

## Abstract

Serpin family E member 1 (SERPINE1) is a serine proteinase inhibitor (serpin) upregulated in diverse types of cancer, including oral squamous cell carcinoma (OSCC), and it functions in an oncogenic role. Hence, exploring pathological mechanism underlying high expression of SERPINE1 is crucial to the targeted therapy of OSCC. Bioinformatics analysis was performed to identify the microRNA (miRNA) and the candidate gene contributing to OSCC progression. The viability, proliferation, and apoptosis of the OSCC cell were evaluated using Cell Counting Kit-8 (CCK-8) assay, BrdU assay, and cell apoptosis assay, respectively. The RNA pulldown assay and luciferase reporter assay were conducted to verify the relationship between SERPINE1 and miRNA 617 (miR-617). SERPINE1 was aberrantly upregulated in OSCC tissues and cell lines. Genetically inhibiting SERPINE1 led to reduction of OSCC cell viability and proliferation and elevation of OSCC cell apoptosis. According to bioinformatics analysis, miR-617 contained a response element for SERPINE1 overexpression, which is validated by the RNA pulldown and luciferase reporter assays. Furthermore, miR-617 was detected to be downregulated in OSCC tissues and cell lines, and it displayed a negative correlation with advanced stages. Besides, miR-617 mimic or inhibitor transfection could suppress or boost the SERPINE1 expression. More importantly, miR-617 mimic could block the effect of SERPINE1 overexpression on OSCC cell proliferation, viability, and apoptosis. SERPINE1 acted as a proproliferative oncogenic factor that is partly regulated by miR-167 downregulation in OSCC cells. Therefore, the miR-617/SERPINE1 axis is a potential therapeutic target against OSCC.

## INTRODUCTION

Head and neck squamous cell carcinoma (HNSCC) refers to a malignancy that develops mainly in the upper aerodigestive epithelium (1). The vast majority of HNSCC cases are linked to oral squamous cell carcinoma (OSCC), which is common among people who smoke tobacco or consume alcoholic products (2). Ranked sixth among all frequently occurring cancer types, OSCC affects more than 350,000 patients worldwide, and the deaths of more than 170,000 individuals across the globe were traced to this cancer in 2018 (2, 3). However, tremendous progress has been made in combating OSCC, including by the application of tumor excision, radiotherapy, chemoradiation therapy, and cetuximab/PD-1 immunotherapy. Despite the gains made in treating OSCC, the 5-year survival rate of this disorder remains poor due to high recurrence and drug resistance (4). In addition, the patients usually turn out to be in late stage of tumorigenesis once diagnosed with OSCC, which also increases the cure difficulty and decreases the survival rates (5, 6). More importantly, researchers have yet to identify the molecular mechanism that facilitates the spread of this malignancy. Hence, it is urgent to understand and explore the molecular mechanism fueling the progression and tumorigenesis of OSCC.

Serpin family E member 1 (SERPINE1) is a member of the serine proteinase inhibitor (serpin) superfamily. This protein-coding gene functions as the principal inhibitor of tissue plasminogen activator (tPA) and urokinase (uPA), and it plays a critical role in the homeostasis of coagulation and fibrinolysis in the body (7, 8). A number of studies have demonstrated the aberrant expression of SERPINE1 in multiple cancers and identified its tumorigenic roles (9). For instance, SERPINE1 was found to be highly expressed in gastric adenocarcinoma (GAC), and it contributed to the proliferation and migration of GAC cells (10). SERPINE1 overexpression has also been reported to promote breast cancer metastasis (11). Besides, several studies have documented the high expression of SERPINE1 in OSCC (11–13). Nonetheless, the regulatory mechanisms of SERPINE1 in OSCC progression have not been systematically explored.

In our present study, we focused on the microRNA (miRNA)-mediated SERPINE1 gene repression in the malignancy of OSCC, since posttranscriptional regulation controlled by miRNAs is well documented in the carcinogenesis of pan-cancers, including OSCC (13–17). We collectively analyzed GEO database records related to OSCC and identified SERPINE1 as one of the most valuable molecules regulating OSCC progression and further verified its oncogenicity via a series of *in vitro* functional assays. We also used bioinformatics analysis to screen novel miRNAs with dysregulated expression responsive to SERPINE1 overexpression in OSCC. Listed as a downregulated miRNA, miRNA 617 (miR-617) was verified in OSCC tissues and cells and proved to target SERPINE1 in the OSCC oncogenesis. These findings deepen understanding of the pathogenesis of OSCC and provide new clues for OSCC clinical treatment.

## RESULTS

### SERPINE1 is the key gene involved in OSCC progression.

**FIG 1:**
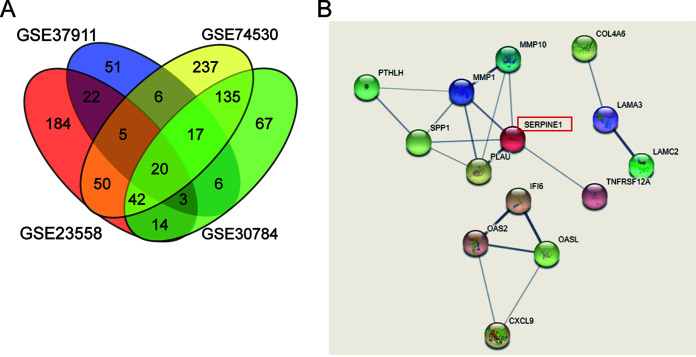
SERPINE1 is a key gene in OSCC. (A) 20 upregulated differentially expressed genes (DEGs) were overlapped from GEO accession numbers GSE37911, GSE74530, GSE30784, and GSE23558. (B) SERPINE1 was the central gene after the protein-protein interaction (PPI) analysis by STRING.

To identify the key gene involved in OSCC progression, four mRNA expression profiles (GEO accession numbers GSE37911, GSE74530, GSE23558, and GSE30784) were downloaded from the GEO DataSets database. With an adjusted *P* (adj.*P*) value of <0.05 and a log_2_ fold change (log_2_FC) of >2, 20 upregulated differentially expressed genes (DEGs) were overlapped (Fig. 1A). After the 20 screened DEGs were then uploaded to STRING for protein-protein interaction (PPI) analysis, it was found that SERPINE1 was the central gene of the PPI network (Fig. 1B). This result suggested the potential role of this protein-coding gene in OSCC.

### SERPINE1 shows aberrantly higher expression in OSCC tissues and cells.

**FIG 2:**
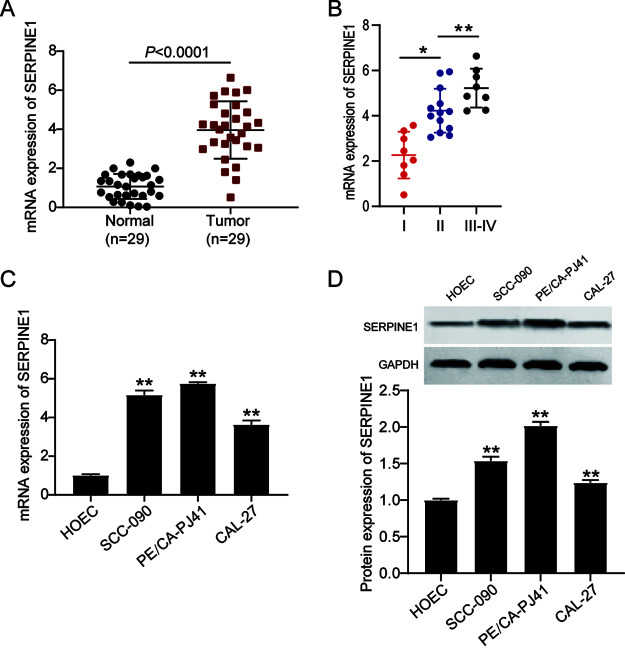
Aberrantly higher expression of SERPINE1 in the OSCC tissues and cells. (A) Reverse transcription-quantitative PCR (RT-qPCR) analysis of the mRNA expression of SERPINE1 in OSCC tissues and adjacent normal oral tissues collected from 29 OSCC patients. (B) SERPINE1 expression increased as the cancer stage advanced in OSCC patients. *, *P* < 0.05; **, *P* < 0.001. (C) RT-qPCR analysis of the mRNA expression of SERPINE1 in OSCC cell lines (SCC-090, PE/CA-PJ41, and CAL-27) and a normal oral epithelial cell line (HOEC). (D) Western blot analysis of the protein levels of SERPINE1 in OSCC cell lines (SCC-090, PE/CA-PJ41, and CAL-27) and a normal oral epithelial cell line (HOEC). Data are presented as the mean ± standard error of the mean (SEM) with three independent experiments. **, *P* < 0.001 compared to HOEC cells.

OSCC tissues and adjacent normal oral tissues from 29 OSCC patients were collected to examine SERPINE1 expression. The reverse transcription-quantitative PCR (RT-qPCR) results indicated a 4-fold increase of SERPINE1 in OSCC tumor tissues compared with adjacent normal oral tissues (Fig. 2A). Besides, higher SERPINE1 mRNA levels were positively associated with tumor-node-metastasis (TNM) staging of OSCC patients (Fig. 2B). Meanwhile, the results of the RT-qPCR analysis in human OSCC cell lines (SCC-090, PE/CA-PJ41, and CAL-27) and in a normal oral epithelial cell line (HOEC) showed that the mRNA level of SERPINE1 was higher in OSCC cell lines than in the HOEC cell line (Fig. 2C). Western blot assay results revealed that SERPINE1 expression increased in OSCC cell lines compared to HOEC cell lines (Fig. 2D). Because of the increases in SCC-090 and PE/CA-PJ41 cell lines, these two cell lines were used in subsequent experiments.

### SERPINE1 silencing inhibits cell proliferation and enhances cell apoptosis.

**FIG 3:**
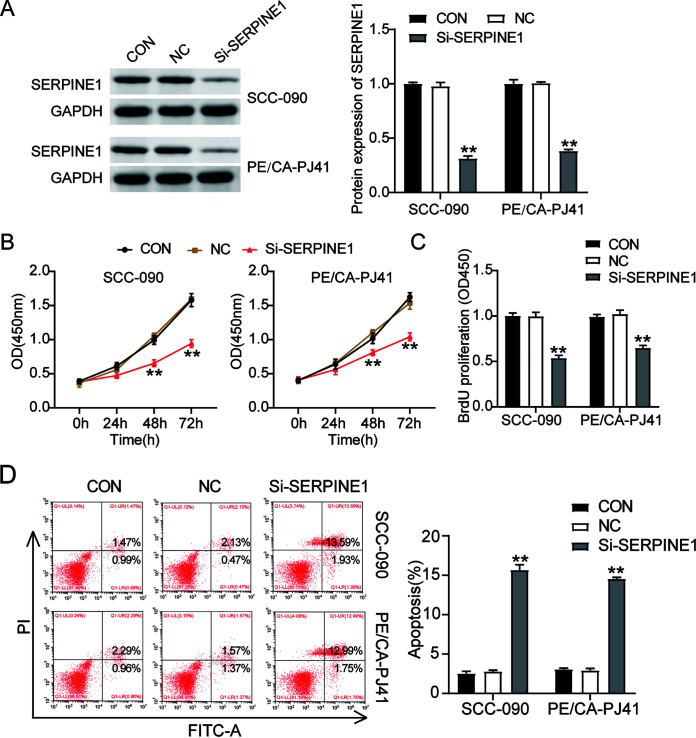
Silencing SERPINE1 inhibits proliferation and enhances apoptosis of OSCC cells. (A) Western blot analysis of the transfection efficiency of SERPINE1 small interfering RNA (si-SERPINE1) in SCC-090 and PE/CA-PJ41 cell lines. (B) The cell viability of SCC-090 and PE/CA-PJ41 cell lines transfected with si-SERPINE1 was determined by CCK-8 assay. (C) The cell proliferation of SCC-090 and, PE/CA-PJ41 cell lines transfected with si-SERPINE1 was examined by BrdU assay. (D) The apoptosis rates of SCC-090 and PE/CA-PJ41 cell lines transfected with si-SERPINE1 were evaluated by cell apoptosis assay. NC, negative control; CON, blank control. **, *P* < 0.001 (compared to CON group). Data are presented as the mean ± SEM with three independent experiments.

SERPINE1 small interfering RNA (si-SERPINE1) was synthesized and transfected into SCC-090 and PE/CA-PJ41 cells to reconstruct SERPINE1-knockdown cells (Fig. 3A). To evaluate the impact of SERPINE1 silencing on OSCC cells, a Cell Counting Kit-8 (CCK-8) assay was performed, and the viability of SCC-090 and PE/CA-PJ41 was observed after transfecting them with si-SERPINE1. The CCK-8 assay results showed that compared to the control group, the viability of OSCC cells in the si-SERPINE1 group was significantly reduced (Fig. 3B). As for the BrdU assay results, the DNA synthesis of SERPINE1 silencing in OSCC cells decreased by 45% in contrast to that of the control group (Fig. 3C), indicating a suppressive effect of SERPINE1 silencing on the proliferation of OSCC cells. However, the outcome of the cell apoptosis assay revealed that SERPINE1 silencing increased the apoptosis rate of OSCC cells by more than 6-fold (Fig. 3D). Collectively, these data suggested that silencing SERPINE1 could inhibit the proliferation of OSCC cells and enhance the apoptosis rate of OSCC cells.

### SERPINE1 is a target gene of miR-617 in OSCC cells.

**FIG 4:**
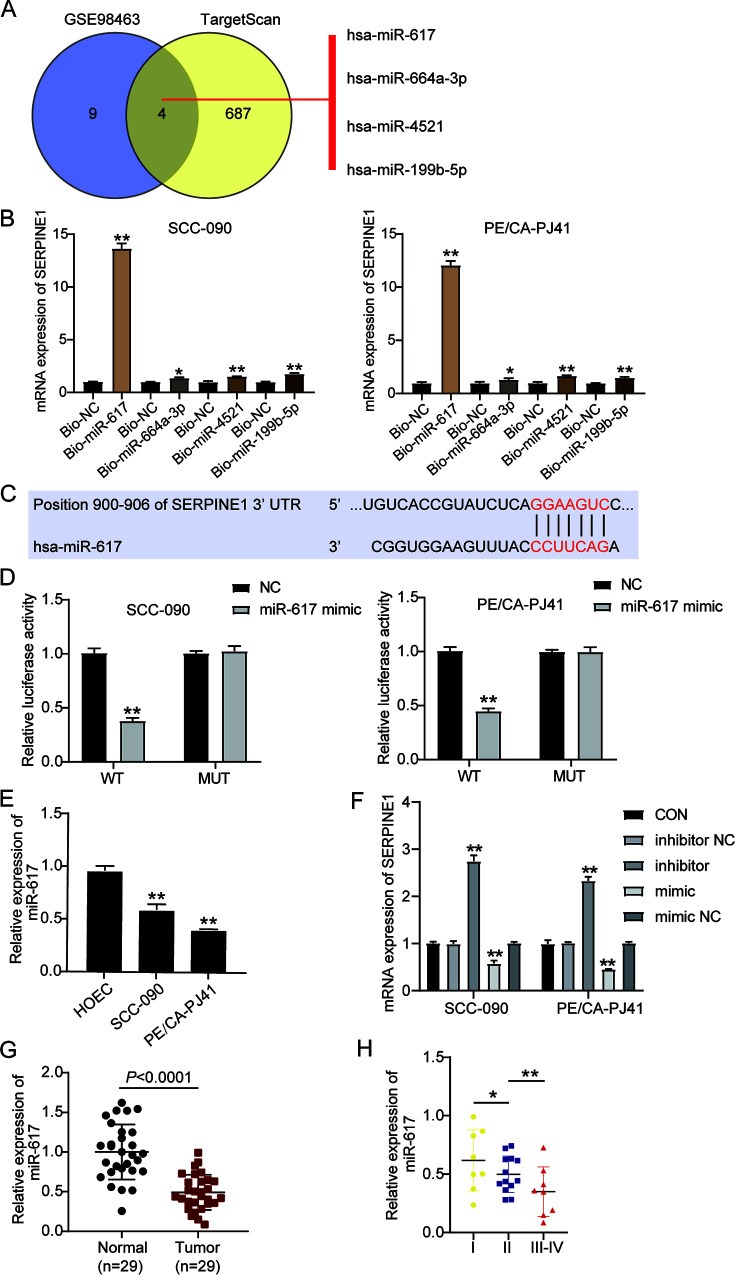
SERPINE1 is a target gene of miR-617 in OSCC cells. (A) Bioinformatics analysis of potential microRNAs (miRNAs) targeting SERPINE1. (B) Transfected cells were subjected to a RNA pulldown assay for determining the interaction between SERPINE1 and four miRNAs (miR-617, miR-664a-3p, miR-4521, and miR-199b-5p). (C) The potential binding sites of miR-617 in the 3′ untranslated region (UTR) of SERPINE1 mRNA predicted by TargetScan 7.0. (D) A luciferase reporter assay was performed to reveal the target relationship between miR-617 and SERPINE1. (E) RT-qPCR analysis of miR-617 expression in OSCC cell lines (SCC-090 and PE/CA-PJ41) and normal a oral epithelial cell line (HOEC). (F) RT-qPCR of SERPINE1 in SCC-090 and PE/CA-PJ41 cell lines transfected with miR-617 inhibitor or mimic and their negative controls (NC). (G) RT-qPCR analysis of miR-617 expression in OSCC tissues and adjacent normal oral tissues collected from 29 OSCC patients. (H) miR-617 expression gradually decreased as cancer stage progressed. NC, negative control; CON, blank control. *, *P* < 0.05; **, *P* < 0.001 (compared to NC group or HOEC group or CON group). Data are presented as the mean ± SEM with three independent experiments.

As gene transcription is associated with the regulation of miRNAs, bioinformatics analysis was performed to identify the potential miRNAs targeting SERPINE1. The results showed four overlapped miRNAs in the databases, namely, miR-617, miR-664a-3p, miR-4521, and miR-199b-5p (Fig. 4A). To confirm their ability to interact with SERPINE1, RNA pulldown assay was performed by transfecting biotinylated miRNA mimics or biotinylated negative control into SCC-090 and PE/CA-PJ41 cells and analyzing the enrichment of SERPINE1. As shown in Fig. 4B, SERPINE1 was mainly enriched in the cells transfected with biotinylated miR-617. Therefore, miR-617 was predicted to be the most valuable miRNA that could target and regulate SERPINE1.The targeted relationship between miR-617 and SERPINE1 was further validated, as illustrated that the miR-617 binding sites in the 3′ untranslated region (UTR) of SERPINE1 mRNA in the Fig. 4C. Next, luciferase reporter plasmids containing wild-type (WT) and mutant (MUT) binding sites of miR-617 were constructed to verify the target relationship between miR-617 and SERPINE1. The data of luciferase assay displayed that the miR-617 mimic reduced the luciferase activity of OSCC cells transfected with WT plasmid by 50%, while no significant change was found in cells transfected with the MUT plasmid (Fig. 4D). Reduced expression of miR-617 was also observed in SCC-090 and PE/CA-PJ41 cells (Fig. 4E). Subsequently, we verified SERPINE1 expression in SCC-090 and PE/CA-PJ41 cells transfected with the miR-617 mimic and miR-617 inhibitor. As shown in Fig. 4F, compared to the miR-617 mimic transfection, the miR-617 inhibitor transfection restored SERPINE1 mRNA levels. Moreover, the expression level of miR-617 was measured in OSCC tissues, and it was found that the expression level of miR-617 in OSCC tissues decreased compared with normal oral tissues (Fig. 4G). The expression was the highest in stage I OSCC subjects and gradually reduced along with TNM staging (Fig. 4H). Collectively, the results indicated that miR-617 could limit SERPINE1 expression by binding its 3′ UTR and that the highly expressed SERPINE1 in OSCC might partly account for the downregulation of miR-617 expression.

### miR-617 influences the proliferation and apoptosis of OSCC cells by targeting SERPINE1.

**FIG 5:**
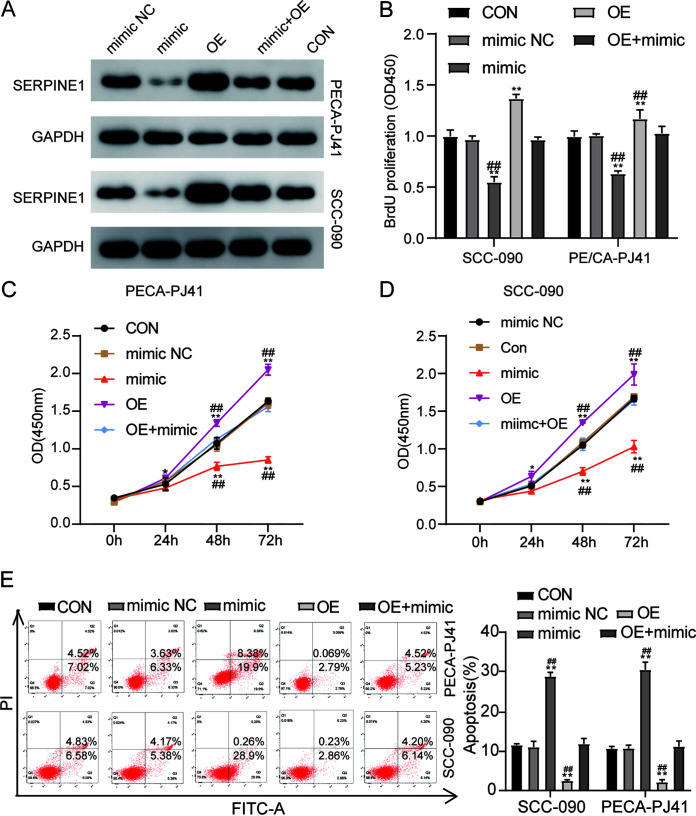
miR-617 acts as a tumor suppressor of OSCC through targeting SERPINE1. (A) Western blot analysis of the protein level of SERPINE1 in SCC-090 and PE/CA-PJ41 cell lines cotransfected with miR-617 mimic and *pcDNA 3.1*-SERPINE1. (B) Cell viability of SCC-090, PE/CA-PJ41 cell lines transfected with *pcDNA 3.1*-SERPINE1 (OE) and/or miR-617 mimic was determined by CCK-8 assay. (C) The cell proliferation of SCC-090 and PE/CA-PJ41 cell lines transfected with si-SERPINE1 and/or miR-617 inhibitor was examined by BrdU assay. (D) The apoptosis rate of SCC-090 and PE/CA-PJ41 cell lines transfected with si-SERPINE1 and/or miR-617 inhibitor was evaluated by cell apoptosis assay. NC, negative control; CON, blank control. **, *P* < 0.001 (compared to CON group); ##, *P* < 0.01 (compared to OE+mimic). Data are presented as the mean ± SEM with three independent experiments.

To evaluate the impact of miR-617 on OSCC development, miR-617 mimic or miR-617 mimic NC was transfected into SCC-090 and PE/CA-PJ41 cells together with *pcDNA 3.1*-SERPINE1 vectors (OE) or its empty vectors (NC). Western blot results showed that the transfection of the miR-617 mimic suppressed SERPINE1 expression caused by OE vector transfection (Fig. 5A). While SERPINE1 ectopic expression facilitated DNA synthesis, the miR-617 mimic reversed this phenomenon (Fig. 5B). Next, a CCK-8 assay was performed to observe the proliferation ability of SCC-090 and PE/CA-PJ41 cells. Findings indicated that, in contrast to the control group, SERPINE1 expression boosted the viability of SCC-090 and PE/CA-PJ41 cells. However, this enhancement effect was completely reversed by cotransfecting SERPINE1-OE vectors (Fig. 5C). In addition, it was discovered that transfecting miR-617 increased the cell apoptosis rate in SCC-090 and PE/CA-PJ41 cells by 4-fold compared to the control group, while could effectively reverse this reduction caused by SERPINE1 ectopic expression to a comparable level to that in the control group (Fig. 5D) These data collectively revealed that the oncogenic properties of SERPINE1 in OSCC might be negatively regulated by miR-617.

## DISCUSSION

Previous investigations have highlighted the prognostic significance of SERPINE1 in OSCC (11–13). However, the underlying mechanism is largely unknown. In this study, we performed bioinformatics analysis and found that SERPINE1 was associated with the progression of OSCC. SERPINE1 expression was detected in OSCC tissues, and it had a positive correlation with an advanced tumor stage. Also, SERPINE1 silencing could suppress OSCC cellular proliferation and trigger OSCC cellular apoptosis. Accumulating studies confirmed that miRNAs could regulate the malignancy of several types of cancers by targeting protein-coding genes (18, 19); however, miR-617 was distinguished by its transcriptional regulation to SERPINE1. Multiple lines of evidence supported this finding; low expression of SERPINE1 was detected in OSCC tissues and cells and was negatively associated with an advanced tumor stage. The results of the RNA pulldown and luciferase assays validated that miR-617 could target SERPINE1 in OSCC. The miR-617 mimic and mimic inhibitor, however, could alter the SERPINE1 mRNA levels in OSCC cells. Meanwhile, the blunted proliferation and induced apoptosis of OSCC cells caused by miR-617 overexpression could be restored by highly expressed SERPINE1.

Previously, the fibrinolytic system was demonstrated to not only regulate the intravascular fibrin deposition but also participate in various pathological processes, such as tumorigenesis (20). It could influence tumor growth and metastasis by regulating angiogenesis and cell migration (21, 22). SERPINE1, one of the major inhibitors of fibrinolysis (7), was explored and was found to play a positive role in the initiation and progression of cancer types, including gastric adenocarcinoma (10), breast cancer (11), ovarian cancer (23), and lung cancer (24). Studies on OSCC also revealed that SERPINE1 was overexpressed in the serum and tissues of patients with OSCC, indicating SERPINE1 played an oncogenic role for OSCC (12, 13, 25). Similarly, we confirmed that SERPINE1 was associated with the occurrence of OSCC and was aberrantly upregulated in OSCC tissues and cells. Furthermore, we discovered that SERPINE1 contributed to the progression of OSCC by promoting OSCC cellular proliferation and limiting their apoptosis. This finding was consistent with the reports of SERPINE1 in other cancer types.

Many studies have explored the effect of miR-617 on several cancer types. This miRNA was first identified in a neoadjuvant chemotherapy study as one of the miRNAs sensitive to chemotherapy in esophageal carcinoma cell lines (26). Years later, miR-617 was found to be associated with the drug resistance of ovarian cancer (16). In this research, we identified the SERPINE1 3′ UTR as containing a miR-617 binding site. By targeting SERPINE1, miR-617 suppressed OSCC cellular proliferation and viability and promoted OSCC cellular apoptosis. Also, miR-617 expression was reduced in OSCC tissues, and it displayed a negative association with the clinical TNM stages of OSCC patients. Also, SERPINE1 mRNA levels were influenced by the miR-617 mimic and miR-617 inhibitor. Hence, miR-617 downregulation might augment the expression of SERPINE1 and amplify the oncogenic function in OSCC.

To our knowledge, this study is the first to describe the role of miR-617 in OSCC. In spite of these important findings, several limitations still exist in this study. For example, the signaling pathway used by SERPINE1 to regulate cell proliferation and apoptosis was not demonstrated. Also, this study did not explore whether SERPINE1 could play a role in the angiogenesis of OSCC. These limitations should be addressed in future studies. Another limitation is that *in vitro* experiments were performed to assess the roles of miR-617 and SERPINE1 in OSCC. Future studies should conduct *in vivo* experiments to validate our findings.

In conclusion, our study suggested that miR-617 could function as a tumor suppressor in OSCC cells by negatively regulating SERPINE1. This knowledge could be useful in finding novel diagnostic and therapeutic targets for OSCC.

## MATERIALS AND METHODS

### Bioinformatics analysis.

GSE37911, GSE74530, GSE23558, and GSE30784 were downloaded from the GEO DataSets database, and they included mRNA expression data involving OSCC. GSE98463 was also downloaded from GEO DataSets, and it included miRNA expression data involving OSCC. With an adjusted *P* value (adj.*P*) of <0.05 and a log_2_ fold change (log_2_FC) of >2, the upregulated differentially expressed genes (DEGs) were distinguished. STRING was then applied to predict the protein-protein interactions (PPI) for the upregulated DEGs. With an adj.*P* value of <0.05 and a log_2_FC of less than −1, downregulated, differentially expressed miRNAs were identified. TargetScan was used to predict the miRNAs binding to our gene of interest.

### Sample collection.

Biopsy specimens of OSCC tissues were taken from 29 patients who underwent tumor resection from November 2018 to January 2020 at the Sixth Hospital of Wuhan. The patients selected in this study had complete medical records and no history of anxiety disorders or other medical complications. The clinical characteristics of the participants are shown in Table 1. Completed informed consent forms were obtained from all recruited patients prior to the sample collection. This study was performed according to the guidelines enshrined in the Declaration of Helsinki and was approved by the Ethical Committee of the Sixth Hospital of Wuhan.

**TABLE 1:** Clinical characteristics of 29 OSCC patients

Clinical characteristic	No. (%) of cases^*a*^
Gender	
Male	17 (58.62)
Female	12 (41.38)
Age (yrs)	
>55	13 (44.83)
≤55	16 (55.17)
Grade	
Well differentiated	12 (41.38)
Moderate differentiated	12 (41.38)
Poor differentiated	5 (17.24)
Stage	
I	8 (27.59)
II	13 (44.83)
III	5 (17.24)
IV	3 (10.34)
Smoking	
Yes	10 (34.48)
No	19 (65.52)
Alcohol	
Yes	13 (44.83)
No	16 (55.17)

a Total *n* = 29.

### Cell culture.

The human normal oral epithelial cell line (HOEC) and three oral squamous cell carcinoma cell lines (SCC-090, PE/CA-PJ41, and CAL-27) were bought from the American Type Culture Collection (ATCC, USA). The cells were maintained in Dulbecco’s modified Eagle’s medium (DMEM; Gibco, USA) supplemented with 10% fetal bovine serum (Gibco, USA), 100 μg/ml streptomycin, and 100 U/ml penicillin in a humidified atmosphere containing 5% CO_2_ at 37°C.

### RNA extraction and RT-qPCR.

Total RNA was extracted from the HOEC or OSCC cell lines and tissues with RNAiso Plus (TaKaRa, Japan) or the mirVana Paris kit (Life Technologies, USA), according to the manufacturer’s guidelines. For mRNA quantification, the PrimeScriptVR RT reagent kit (TaKaRa, Japan) was utilized to reverse transcribe the RNA extracted with RNAiso Plus to cDNA. After that, the RNA was subjected to specific primer-based reverse transcription-quantitative PCR (RT-qPCR) using SYBR Premix *Ex Taq* (TaKaRa, Japan). For miRNA quantification, the total RNA extracted with the miRVana Paris kit was reverse transcribed and quantified with the All-in-One miRNA RT-qPCR detection kit (GeneCopoeia, China). After that, RT-qPCR was performed with the 7900HT Fast real-time PCR system (Thermo Fisher Scientific, USA). Subsequently, the threshold cycle (2^−ΔΔ^*^CT^*) method was utilized to calculate relative expression of RNA, with GAPDH and U6 as the mRNA internal control and the miRNA internal control, respectively. Table 2 indicates the primer sequences used in this study.

**TABLE 2:** Primer sequences for RT-qPCR

Gene	Primer sequence
SERPINE1	Forward: 5′-GCTTACAGGAGCTTTTGTGT-3′
	Reverse: 5′-ACTCTGAGATGAAAGGGTGTTT-3′
miR-617	Forward: 5′-GCCGAGAGACTTCCCATTTGA-3′
	Reverse: 5′-CTCAACTGGTGTCGTGGA-3′
GAPDH	Forward: 5′-TTGATTTTGGAGGGATCTCG-3′
	Reverse: 5′-CAATGACCCCTTCATTGACC-3′
U6	Forward: 5′-GTGCTCGCTTCGGCAGCA-3′
	Reverse: 5′-CAAAATATGGAACGCTTC-3′

### Western blot assessment.

The total protein was extracted from the HOEC cell line or OSCC cell lines using radioimmunoprecipitation assay (RIPA) lysis buffer and was quantified with the Pierce BCA protein assay kit (Thermo Fisher Scientific, USA). After that, 30 μg extracted protein was loaded into an 10% SDS-PAGE gel and separated by electrophoresis. Next, the protein bands were electrotransferred onto a polyvinylidene difluoride (PVDF) membrane (Millipore, USA) and washed three times with PBST (0.1% Tween 20–phosphate-buffered saline [PBS] [vol/vol]). The membrane was then blocked with 5% bovine serum albumin (BSA) for 2 h at room temperature, followed by primary antibody incubation overnight at 4°C. After washing the membrane three times with PBST, it was incubated in diluted secondary antibody at room temperature for 1.5 h. Finally, the blots were visualized with the Enhanced Chemiluminescent reagent kit (Thermo Fisher Scientific. USA). The primary antibodies used were rabbit monoclonal anti-SERPINE1 (1:1,000, catalog no. ab222754; Abcam, USA) and mouse monoclonal anti-GAPDH (1:1,000, catalog no. ab8245; Abcam, USA). The secondary antibodies were horseradish peroxidase (HRP)-conjugated goat anti-rabbit IgG(H+L) (1:1,000, catalog no. ab205718; Abcam, USA) and HRP-conjugated goat anti-mouse IgG(H+L) (1:1,000; catalog no. ab6789; Abcam, USA).

### Cell transfection.

The SCC-090 and PE/CA-PJ41 cell line in the logarithmic phase was first trypsin digested and then seeded into 6-well plates at a density of 8 × 10^5^ cells/well. After overnight incubation at 37°C with 5% CO_2_, the cells were transiently transfected with 50 nM SERPINE1 siRNA(si-SERPINE1) or *pcDNA 3.1*-SERPINE1 (OE), miR-617 inhibitor, miR-617 mimic, or corresponding negative controls (NC). This transfection was done with Lipofectamine 3000 reagent (Invitrogen, USA). At 48 h posttransfection, the transfected cells were collected for further assays. The si-SERPINE1, *pcDNA 3.1*-SERPINE1 (OE), miR-617 inhibitor, miR-617 mimic, and negative controls were obtained from RiboBio, China.

### CCK-8 assay.

The Cell Counting Kit-8 (CCK-8) (Yeasen, China) was employed to determine the viability of SCC-090 and PE/CA-PJ41 cell lines according to the manufacturer’s instructions. Briefly, the SCC-090 and PE/CA-PJ41 cells transfected were collected and seeded in 96-well plates at a density of 3 × 10^3^ cells/well. After incubation for 0 h, 24 h, 48 h, and 72 h at 37°C with 5% CO_2_, 10 μl CCK-8 solution was added, and the cells were incubated for another 2 h. Finally, the absorbance at 450 nm was measured with a microplate reader (Bio-Rad, USA).

### BrdU assay.

The proliferation ability of SCC-090 and PE/CA-PJ41 cells was evaluated with the BrdU cell proliferation assay kit (CST, USA). The transfected cells were harvested and trypsin digested before being added to a 96-well plate at a density of 5 × 10^3^ cells/well. After culturing the cells for 24 h, 10 μl of 10× BrdU labeling solution was added to incorporate BrdU into the cell DNA, and the mixture was incubated for 24 h. After the medium was removed and the fixing/denaturing solution (100 μl/well) was added for 30 min, the cells were incubated at room temperature. The medium was then replaced by 100 μl prepared 1× detection antibody solution, and the mixture was incubated for 1 h at room temperature. After the cells were removed and washed three times using 1× wash buffer, they were incubated with 100 μl 1× HRP-conjugated secondary antibody solution at room temperature for 30 min. Next, 100 μl 3,3′,5,5′-tetramethyl benzidine (TMB) substrate was added to each well for 30 min, and the mixture was incubated at room temperature. Finally, 100 μl stop solution was added, and the absorbance at 450 nm was measured with a microplate reader (Bio-Rad, USA).

### Cell apoptosis assay.

The apoptosis of SCC-090 and PE/CA-PJ41 cells was determined by performing flow cytometry with the fluorescein isothiocyanate (FITC)-annexin V apoptosis detection kit I (BD Biosciences, USA). First, 2 × 10^6^ transfected SCC-090 and PE/CA-PJ41 cells were collected and washed twice with cold PBS. After that, they were resuspended in 100 μl prepared 1× binding buffer. Next, 5 μl FITC annexin V and 5 μl propidium iodide (PI) staining solution were added to the cell suspension, and the mixture was incubated in the dark at 25°C for 15 min. Finally, 400 μl prepared 1× binding buffer were supplemented, and cells were loaded into the BD FACSCalibur flow cytometry system (BD Biosciences, USA) to analyze the cell apoptosis rate.

### RNA pulldown assay.

In this assay, SCC-090 and PE/CA-PJ41 cells (6 × 10^5^) were first seeded into a 6-well plate and cultured for ∼18 to 24 h at 37°C in air containing 5% CO_2_. Next, the biotinylated miRNA mimics or biotinylated negative control (RiboBio, China) were transiently transfected into SCC-090 and PE/CA-PJ41 cells using Lipofectamine 3000 reagent (Invitrogen, USA). After being cultured for 48 h, the cells were harvested and lysed to obtain cell lysates. The lysates were then sonicated and incubated at 4°C for 3 h with streptavidin beads (Life Technologies, USA). Subsequently, the streptavidin beads were collected by centrifugation and washed twice with PBS. The RNAs attached to the streptavidin beads were then eluted by using the RNeasy minikit (Qiagen). They were eventually subjected to RT-qPCR to measure the relative expression of SERPINE1.

### Luciferase reporter assay.

The wild-type (WT) and mutant (MUT) reporter plasmids were first constructed by cloning the wild-type or mutant 3′ UTR of the human SERPINE1 mRNA 3′ UTR into psiCHECK-2 (a luciferase reporter plasmid). The wild-type or mutant luciferase reporter plasmids, together with miR-617 mimics or negative controls (NC), were subsequently cotransfected into SCC-090 and PE/CA-PJ41 cells using Lipofectamine 3000 reagent (Invitrogen, USA). After that, the mixture was incubated for 48 h in an atmosphere containing 5% CO_2_ at 37°C. Next, the transfected cells were collected and lysed into the cell lysates. The lysates were finally subjected to a dual-luciferase reporter assay system (Promega, USA).

### Statistical analysis.

The data collected in this study consisted of three biological repeats, and they were expressed as mean ± standard deviation (SD). Prism 8.0 (GraphPad Software, USA) was employed to perform statistical analysis and statistical graph construction. The Student’s *t* test and one-way analysis of variance (ANOVA) with Dunnett’s *post hoc* test were applied to compare the statistical differences between two groups and multiple groups, respectively. *P* values of less than 0.05 were considered statistically significant.
